# Uganda’s disability journey: Progress and challenges

**DOI:** 10.4102/ajod.v3i1.108

**Published:** 2014-11-25

**Authors:** Julie Abimanyi-Ochom, Hasheem Mannan

**Affiliations:** 1 Deakin Health Economics, Population Health SRC, Deakin University, Australia; 2 Nossal Institute for Global Health, The University of Melbourne, Australia

## Introduction

The International Classification of Functioning, Disability and Health (ICF) defines disability as a complex phenomenon, reflecting the interaction between features of a person’s body and features of the society in which he or she lives (World Health Organization [WHO] 2002:2). Based on the ICF definition of disability, over a billion people worldwide and 19% of the Ugandan population are estimated to have some form of disability (Uganda Bureau of Statistics and ICF International 2012:27; World Health Organization & World Bank 2011:ix). The prevalence of disability is predicated to increase in the future due to ageing populations and an increase in chronic health conditions hence the need to urgently deal with global disability (World Health Organization & World Bank 2011:ix).

Uganda has been praised as one of the champions in sub-Saharan Africa for advocating for the rights of persons with disabilities (Katsui & Kumpuvuori 2008; Lang & Murangira 2009:18–24), with their rights incorporated in the national legal framework. This includes the 1995 constitution which recognises the rights of persons with disabilities to attain full mental and physical potential as well as development of the 2006 National Policy on Disability. Several laws have been established in the national legal framework to advocate for the rights of people with disabilities (PWDs) as highlighted below:

- The 1996 Children’s Statute for early assessment of disabilities amongst children to achieve early treatment, rehabilitation and education.
- The Parliamentary Elections Statute of 1996 established five positions in parliament of which one of them must be a woman, and recognised the use of sign language for the deaf in parliament (International Labour Organization [ILO] 2004:6; Republic of Uganda 2006:12).
- The 1997 Local Government Act that established representation of PWDs at all local government levels for both males and females.
- The 1997 Uganda Communication Act for the development of techniques and technologies to ensure communication services for PWDs and the 1997 Universal Primary Education Act which demands families to give CWDs priority at enrolment.
- The 1998 UNISE Act, for the establishment of the Uganda National Institute of Education (UNISE) for special teacher training for children with disabilities (CWDs).
- Others include the special allocation of university scholarships for persons with disabilities through affirmative action and the right to assets including land (Hanass-Hancock & Nixon 2009; Katsui & Kumpuvuori 2008; Lang & Murangira 2009:17; Republic of Uganda 2006:11–12).

Internationally, Uganda is a signatory to several international pieces of legislation advocating for the rights of persons with disabilities including the 2008 United Nations Convention on the Rights of Persons with Disabilities and 1983 International Labour Organisation Convention on Vocational Rehabilitation and Employment of disabled persons (ILO 2004:8; Lang & Murangira 2009:5).

### Progress to date

The practical enactment of the aforementioned laws include the election of PWDs at all levels of political life from the village to parliament, making Uganda one of the countries with the highest numbers of elected representatives with a disability in the world (World Health Organization & World Bank 2011:171). Also, UNISE, an institute of higher learning with specialised programmes to address professional teacher development was established in 1991 by the government of Uganda (ILO 2004:10; Kyambogo University 2014). UNISE trains teachers and community workers to support and work with PWDs including children with disabilities (ILO 2004:10; Kyambogo University 2014).

Uganda’s commitment to providing education to children with disabilities dates back to a modest start in 1983 when a one-staff section for special needs education was established in the Ministry of Education. In 1987 the government established the ‘Kajubi Commission’ to review the entire education sector, and its report of 1989 emphasised the need for government to prioritise special needs education a recommendation which was adopted in the 1992 government White Paper on Education. According to the Ministry of Education and Sports, in 2008, there were 183 537 learners with disability in primary schools countrywide, and 11 145 learners in secondary schools countrywide (Foundation for Human Rights Initiative 2009).

Uganda’s initial report to the Committee on the United Nations Convention on the Rights of Persons with Disabilities (UNCRPD 2010) reports that:

all government programs for promoting education – Universal Primary education (UPE), Universal Secondary Education (USE) and Business and Vocational Technical Training are all embedded with affirmative action for learners with disabilities. *The Business, Technical, Vocational Education and Training (BTVET) Act, No. 12* of 2008, promotes equitable access to education and training for all disadvantaged groups, including disabled people. Uganda promotes both inclusive education and special needs education where it is needed, all the 21 000 schools in Uganda practice Inclusive Education by admitting learners with special Education needs. (p. 35)

In addition, Uganda has a strong training focus on Community Based Rehabilitation (CBR) programmes, established in 1992 under the Ministry of Gender, Labour and Social Development with assistance from the Norwegian Association of the Disabled (NAD). CBR follows WHO strategy for involving PWDs in developing their communities through equal access to community resources including health, education, rehabilitation and employment, and ensure social inclusion of PWDs. A five year National CBR Strategic Plan 2002–2007 was developed to fully integrate PWDs into the community and ensure equal opportunities for PWDs. Therefore, PWDs and local communities have been empowered to manage disabilities, identify children with special needs and increase access to education facilities; for example, through the Alternative Basic Education for Karamoja (ABEK) programme (ILO 2004:9–10; Norwegian Agency for Development Cooperation [Norad] 2011:110). Through the CBR programme, 80% of the PWDs are helped within the community whilst the rest require specialist services. CBR programme uses a multisectoral approach and the main activities include capacity building, economic empowerment, increasing disability awareness, disability management and home based care (Claussen, Kandyomunda & Jareg 2005; Norad 2011:87).

The disability movement in Uganda has been spearheaded by the National Union of Disabled Persons Uganda (NUDIPU), established in 1987. NUDIPU represents all disability groups in Uganda including women with an objective of advocating for equal opportunities and involvement of PWDs in policy development and implementation of programmes addressing disability. This is usually in collaboration with other stakeholders including government and NGOs (ILO 2004:12); for example, PWDs have been involved in the third phase of the Poverty Reduction Strategy Papers/Poverty Eradication Action Plan (PRSPs/PEAP) process which is important for inclusion of prodisability poverty alleviation strategies (Dube 2005:28; Norad 2012:33). Likewise, female-specific disability groups managed by women were established including the National Union of Women with Disabilities of Uganda (NUWODU) and the Disabled Women Network and Resource Organisation (DWNRO). The groups train women and advocate for economic empowerment of women with disabilities including access to micro-credit programmes (ILO 2004:12). NUDIPU has been internationally active within the East African region providing advice to disability groups in countries affected by war including Somalia, Sudan, Rwanda and the Democratic Republic of Congo (Lang & Murangira 2009:25).

Comparable to other developing countries, Uganda lacks disability data for monitoring and evaluating disability interventions’ policy. The Uganda Demographic and Health Surveys (UDHS) funded by USAID has been used as an alternative avenue through which data on disability can be improved especially with the recent inclusion of the Washington Group’s Short Set of six questions on disability (Madans, Loeb & Altman 2011; Uganda Bureau of Statistics and ICF International 2012:7). The UDHS is a population sample survey undertaken every four years for monitoring and impact evaluation of population, health, HIV and/or AIDS and nutrition programmes (Measure DHS 2014). The inclusion of disability question in the 2011 UDHS provides opportunities for good data collection and more regular reporting which makes benchmarking disability progress possible. This is important in improving disability data including attainment of consistency in its measurement.

Furthermore, it is essential for international comparison with other developing countries that also include these questions (Mitra 2013; World Bank 2009). Uganda’s commitment to disability is also evidenced through the introduction of programmes targeted at improving socio-economic opportunities of vulnerable populations including PWDs. This includes the special disability grant to support socio-economic development and employment opportunities for PWDs in districts estimated at 12 000 USD per annum (Norad 2012). The Social Assistance Grants for Empowerment (SAGE), a pilot social cash transfer scheme is another similar programme under the Ugandan government’s Expanding Social Protection Programs (ESPP). The programme addresses chronic poverty and aims at improving access to health care, education and other key services for chronically poor people. The evaluation of SAGE revealed that eligible households had a higher proportion of people defined as chronically ill or disabled than noneligible households, with 33% of eligible households containing a chronically ill or disabled member (Oxford Policy Management, Economic Policy Research Centre & Neema 2013:14–15).

### Review of challenges in Uganda’s disability journey

Despite Uganda’s achievements in disability, there are several challenges to its disability journey. There are differing statistics on the prevalence of disability in Uganda mostly due to the use of different definitions of disability and improvement in data gathering methods (Tsitsi *et al*. 2011:6). This makes targeted disability interventions difficult (Lang & Murangira 2009:16). In addition, disability data is quite limited and mostly at the national level (Government of Uganda 2010:54; Lang & Murangira 2009:9). A lack of data, especially at the district and local levels, compounds the difficulties in planning and targeting of services for disability (Lang & Murangira 2009). Moreover, the limited existing data have not meaningfully influenced decisions to include disability in the national development process (Tsitsi *et al*. 2011). As highlighted by Mitra (2013), there is a need to revolutionise disability related data worldwide to attain consistent and reliable disability statistics, which would also be relevant to Uganda.

The national disability policy also indicates research as one of the interventions to improve the limited knowledge on aspects of persons with disabilities through collection of comprehensive information on persons with disabilities. This stipulates that disability data would be collected in national surveys and censuses and disability management information systems developed (Republic of Uganda 2006:21). However, research that focusses on disability is still quite limited (Norad 2012:19); this situation is compounded by the fact that national surveys and census – the proposed avenue for such data collection – may be delayed in some instances (Groce *et al*. 2013; Ninsiima 2013; Uganda Bureau of Statistics 2011). The national census survey is supposed to be undertaken every 10 years, the last of which was in 2002. This makes monitoring and targeting of disability related goals inefficient given the extensive time lag in such surveys and the rapid changes in living conditions of persons with disabilities in all domains of life and across lifespans (Hoogeveen 2005; Loeb, Eide & Mont 2008). In their initial report to the UNCRPD (2010), the Ugandan government acknowledged the lack of authentic statistics as a challenge. They stated that:

[*T*]here are no readily available statistics desegregated by gender, disability and region to back up most of the efforts government has made towards the promotion of the rights of persons with disabilities. (p. 6)

Collecting data on disability more frequently would enable progressive follow-up which would indicate changes in regard to inequities in access to health, education and employment and other domains of life compared to the general population. Nonetheless, analysis of data from census surveys is limited to disability prevalence unless commissioned researchers are contracted to undertake a more elaborate and extensive analysis on disability. There is a need to make collection of disability data and analysis of disability mainstreamed and routine in national reports including Uganda Bureau of Statics, similar to gender analysis.

Uganda’s development policy is consistent with international policies where development programmes, such as the Millennium Development Goals (MDGs), exclude disability; for example, the National Development Plan 2010/11– 2014/15 which succeeded PEAP does not specifically mention disability although disability organisations were consulted as part of the wider civil society consultations (Norad 2012:11). Consequently, the link between disability and development has not been highly prioritised in national development strategies despite evidence of the link between disability and poverty (Hoogeveen 2005; World Bank 2009:131). A Ugandan study by Lwanga-Ntale (2003) reported that 80% of PWDs living in long term poverty with limited access to education, health facilities, sustainable housing and employment. Similarly, a study by Groce *et al*. (2011) revealed that Ugandan households with PWDs were more likely to be poorer than similar households without disabled members. Therefore, the attainment of the MDGs especially education attainment and improvement of health outcomes may not be achieved if Uganda does not make disability a main concern in its development strategies including public health interventions (Mitra & Sambamoorthi 2013; World Health Organization & World Bank 2011:12). This is a future threat especially given that 77% of the Ugandan population is under 30 years old and that 45% of the disability population is under 30 years old (Daumerie & Madsen 2010:5; Lang & Murangira 2009:16).

The human rights based approach to disability in Uganda highlights the importance of attaining the full potential of persons with disabilities in both physical and mental terms. However, implementation of interventions that would ensure this has been slow or non-existent. Research in Uganda has indicated limited use of accessible information especially within education and health service provision (Action on Disability and Development Commissioned Study 2005; Lang & Murangira 2009). Examples of accessible information include sign language, and the use of electronic communications aids that allow the user to picture symbols, letters, and/or words and phrases to create messages (American Speech-Language-Hearing Association 2014). Within the education sector, most mainstream schools do not provide accessible information and persons with disabilities admitted to higher education institutions may have to pay out of pocket for any communication service they need (Foundation for Human Rights Initiative 2009; Murray n.d.). A lack of accessible information in schools has been indicated as one of the reasons for high dropouts in school and poor literacy rates for persons with disabilities (Lang & Murangira 2009; World Bank 2009), resulting in limited employment opportunities. The limited number of accredited educational institutions for training in accessible information exacerbates the problem, making it impossible to meet the demand for such services in Uganda; as a result, children with disabilities (CWDs) lag behind (Republic of Uganda 2006). Likewise, ensuring adequate availability of assistive technologies for mobility is important since such technologies have been found to create greater community participation in Uganda, especially in education and employment (Hunt *et al*. 2004).

On the other hand, government efforts towards inclusive education have often been criticised and accused of putting children with disabilities amongst able bodied children without adequate modifications to the teaching and learning environment, and with inadequate specialised teachers (Foundation for Human Rights Initiative 2009:15, 19). Similarly, when universal primary education was first introduced, there was a large influx of previously excluded groups of children, including those with disabilities. With few additional resources schools were overwhelmed and reported problems with discipline, performance, and drop-out rates amongst students (World Health Organization & World Bank 2011).

In the health sector, the majority of health promotion campaigns and services, including national programmes seldom include accessible information like sign language, braille or audio format (Action on Disability and Development Commissioned Study 2005; United Nations Human Rights, World Health Organization and United Nations Programme on HIV/AIDS 2009:3). In some instances, health centres are not accessible to physically disabled people who face physical and attitudinal barriers in accessing care, such as antenatal services for women (Groce *et al*. 2013). This is further compounded by disability related stigma^1^ and misconceptions by most health service providers (particularly for HIV related services) where persons with disabilities are presumed not to be sexually active (United Nations Human Rights, World Health Organization and United Nations Programme on HIV/AIDS 2009:3; Yousafzai *et al*. 2005). Nonetheless, persons with disabilities have been shown to have greater HIV risk given vulnerability to sexual violence and low literacy levels that limit their knowledge on health related issues including HIV and/or AIDS (Chireshe, Rutondoki & Ojwang 2010; United Nations Human Rights, World Health Organization and United Nations Programme on HIV/AIDS 2009:3).

Uganda’s national policy on disability does not explicitly elaborate on how interventions relating to disability would be funded but does mention the fact that disability is multisectoral and hence the need of each sector to deal with disability in its area of mandate and focus (Republic of Uganda 2006). This makes commitment to disability interventions difficult, leading to lack of coordination between the different ministries concerning disability (Lang & Murangira 2009; Republic of Uganda 2006). Consequently, care and support of persons with disabilities in Uganda is still poor and this has been made worse by the lack of a national social assistance programme specifically for people with disabilities (Chireshe *et al*. 2010). However, similar to gender equity in health and education systems, funds need to be earmarked to disability for interventions to effectively address inequities due to disability. This would require disability specific budgeting across government departments (Payne 2009). As recommended by Norad (2012), there is a need to demand disability disaggregated indicators in all sectors of government planning and reporting as disability is a cross-cutting issue.

Despite Uganda’s active history of disability activism and legislation specific to disability, societal and cultural negative attitudes and perceptions have been indicated as the greatest obstacle to disability inclusion. Such negative attitudes have been reported to prevent genuine consideration of disability within the national development agenda including the PRSPs (Tsitsi *et al*. 2011). Although Uganda has excelled in developing a comprehensive body of legislation that uphold disability rights particularly through affirmative action, many of these have not been implemented. The government’s commitment to disability compared to other cross-cutting issues like gender remains low in terms of implementation. A report by Norad revealed that implementation of a majority of targeted and mainstreamed interventions categorised persons with severe cognitive or intellectual disabilities as ‘all disabilities’, marginalising these individuals within most projects (Norad 2012).

### Comparison with global trends

Uganda joined the global community in rallying together to ensure the improvement of conditions for disadvantaged people in the world through the MDGs. However, none of the eight MDGs, MDG targets indicators or millennium declaration mention people with disabilities despite the fact that persons with disabilities lack equitable access to resources including health, education, work and social and legal systems globally (Chataika *et al*. 2011; Republic of Uganda 2006; United Nations 2011; World Health Organization & World Bank 2011; Yousafzai *et al*. 2005). Such barriers lead to poor economic participation and worse educational outcomes for PWDs, making them more vulnerable (Lang & Murangira 2009; Mitra & Sambamoorthi 2013; United Nations 2011; World Bank 2009; World Health Organization & World Bank 2011). The exclusion of persons with disabilities in MDGs represent a lost opportunity to tackle the social, educational, health and economic problems faced by marginalised persons with disabilities. There is a growing opinion that the MDGs will not be realised unless persons with disabilities are included (United Nations 2011). As a result, there is a push to seek disaggregated disability data for each of the post-2015 MDGs (United Nations 2011, 2013). Such reports will present an opportunity to monitor the progressive realisation of the rights of PWDs globally.

Similar to Uganda, there is evidence of data related challenges globally (Mitra 2013; World Health Organization & World Bank 2011): ‘Appropriate statistical and research data needs to be collected at both country and international levels to assist the CRPD formulate and implement policies to achieve internationally agreed development goals’. This calls for improvement of data at both the national and international level in order to capture all aspects of disability including contextual factors to give a complete picture of disability and functioning. It is advised to disaggregate data further by gender, age, income or occupation to uncover trends, patterns and other information about ‘subgroups’ of people experiencing disability. Furthermore, setting international standards on data and using standardised questions can improve harmonisation and ensure comparison with other countries (Mitra 2013; World Health Organization & World Bank 2011). Therefore, data collected at the national level need to be relevant and comparable at the global level, possibly by basing design on international standards, for example, the International Classification of Functioning, Disability and Health or ICF (Mitra 2013; World Health Organization & World Bank 2011).

## Conclusion

Uganda has excelled in its commitment to disability rights by establishing a comprehensive body of legislation, policies and socio-economic programmes consistent with social justice. The evidence presented in this article highlights there continues to be a gap between laws, policies and practice. The implementation gap is about negative cultural attitudes towards disability, poor funding, inadequate training in inclusive education and limited access to accessible information and assistive mobility devices. The implementation gap makes monitoring progress difficult and discourages prioritising resource allocation to disability (Lang & Murangira 2009; Uganda Bureau of Statistics 2011). Most importantly, there is a lack of benchmarking of policies and indicators of equity in access to health, education, and social protection for persons with disabilities. As Uganda strives towards progressive realisation of rights of persons with disabilities the following recommendations are likely to make a difference in transforming legal and policy commitments into measures of equity:

- Community advocacy to change societal and cultural negative attitudes towards PWDs.
- Training more educators in inclusive education to meet the increased demand in schools due to universal education
- Provision of accessible information and assistive devices to enhance mobility, especially in health and education programmes to ensure social inclusion of PWDs.
- Earmarking of disability funds in all government departments to ensure that disability as a crosscutting issue is prioritised in all government programmes.
- Routine collection of systematic data on disability, mainstreaming of disability in all government reports and extensive analysis of disability data, similar to gender analysis.

Better data collection across all government departments on disability enables performance and policies to be assessed over time.

## Footnotes

1 Women face double stigma for being disabled and for being female in a patriarchal dominated culture like Uganda.
